# The Intricate Crosstalk Between Insulin and Pancreatic Ductal Adenocarcinoma: A Review From Clinical to Molecular

**DOI:** 10.3389/fcell.2022.844028

**Published:** 2022-02-17

**Authors:** Junyuan Deng, Yujie Guo, Jiali Du, Jichun Gu, Lei Kong, Boan Tao, Ji Li, Deliang Fu

**Affiliations:** Department of Pancreatic Surgery, Pancreatic Disease Institute, Huashan Hospital, Fudan University, Shanghai, China

**Keywords:** Insulin, diabetes mellitus, cancer metabolism, pancreatic ductal adenocarcinoma, hyperinsulinemia

## Abstract

Increased insulin level (or “hyperinsulinemia”) is a common phenomenon in pancreatic ductal adenocarcinoma (PDA) patients and signals poor clinical outcomes. Insulin is safe in low PDA risk population, while insulin significantly promotes PDA risk in high PDA risk population. The correlation between insulin and PDA is a reciprocal self-reinforcing relationship. On the one hand, pancreatic cancer cells synthesize multiple molecules to cause elevated peripheral insulin resistance, thus enhancing hyperinsulinemia. On the other hand, insulin promotes pancreatic cancer initiation and sustains PDA development by eliciting tumorigenic inflammation, regulating lipid and glucose metabolic reprogram, overcoming apoptosis through the crosstalk with IGF-1, stimulating cancer metastasis, and activating tumor microenvironment formation (inflammation, fibrosis, and angiogenesis). Currently, taking glucose sensitizing agents, including metformin, SGLT-2 inhibitor, and GLP-1 agonist, is an effective way of lowering insulin levels and controlling PDA development at the same time. In the future, new drugs targeting insulin-related signal pathways may pave a novel way for suppressing PDA initiation and progression.

## Introduction

Pancreatic ductal adenocarcinoma (PDA), or pancreatic cancer, is a devastating exogenous pancreatic disease that ranks the fourth leading cause of cancer mortality and is projected to be the second leading cause of death by 2030 in the United States (Rahib et al., 2014; Siegel et al., 2020). PDA patients often have a dismal fate that only 10% of them could survive longer than 5 years. At present, over 80% of pancreatic cancer cases are in the unresectable tumor stage when diagnosed with extensive local invasion and metastases to distant organs (such as liver, lung, bone, etc.) (Klaiber et al., 2018).

Due to the high prevalence of prediabetic hyperglycemia and diabetes mellitus (DM) in pancreatic cancer patients (over 75% in high glucose status and 50% in diabetic status), a peripheral increase of insulin level and exogenous insulin injection can often be seen in PDA cases (Cetin et al., 2002; Korc, 2007; Dugnani et al., 2016; Andersen et al., 2017; Dev et al., 2018). In prediabetic status, increased blood glucose level stimulates the pancreatic islets to produce more insulin, leading to endogenous hyperinsulinemia in the human body. This compensatory insulin overexpression was gradually decreased with the apoptosis caused by hyperglycemia in pancreatic islet beta cells. Finally, long-standing diabetes patients require an exogenous insulin injection to sustain high-insulin concentration, which is the so-called “exogenous hyperinsulinemia.” Moreover, a unique portal structure enables insulin secreted from the pancreatic islet to perfuse the exocrine pancreas first, significantly increasing the regional insulin concentration in PDA tissue. As a result, pancreatic cancer is often exposed to a high insulin concentration environment. In the past decades, it has been generally accepted that insulin was a risk factor in many types of cancer, including colorectal cancer, breast cancer, liver cancer, and pancreatic cancer (Pollak, 2008). In PDA cases, hyperinsulinemia or exogenous insulin use is related to the elevated PDA incidence and increased tumor volume (Stolzenberg-Solomon et al., 2005; Zyromski et al., 2009; White et al., 2010; Wolpin et al., 2013; Boursi et al., 2017; Carreras-Torres et al., 2017). Thus, insulin has been considered decisive in developing PDA. However, the failure of clinical trials targeting insulin and its related signal pathway indicates there is still lacks a systemic understanding of how insulin interferes with PDA development. And, as a malignant disease initiating from the pancreas, it is not clear how pancreatic cancer influences insulin production and synthesis in the pancreas. A clear elucidation of the crosstalk between insulin and pancreatic cancer is needed at present.

This review summarizes the published clinical and experimental evidence of how insulin interacts with PDA to promote hyperinsulinemia, PDA initiation, and progression. We also concluded the current ways of lowering insulin levels and clinical advances targeting the insulin signal pathway in pancreatic cancer.

## PDA Risk in Diabetic Insulin Users

The peculiar correlation between diabetes and pancreatic cancer was observed as early as 1833 (Iqbal et al., 2013). Diabetes mellitus develops pancreatic cancer, while pancreatic cancer also strengthens hyperglycemic status. According to the diagnostic interval between type 2 diabetes mellitus (T2DM) and PDA, DM was divided as the long-standing diabetes mellitus (time interval longer than 3 years) and the new-onset diabetes mellitus (time interval shorter than 3 years) (Jeon et al., 2018). PDA risk is slightly increased in the LSDM group compared with the euglycemic group. However, LSDM is considered to be a relatively low PDA risk group because PDA was reported to have no causal relationship with LSDM(Molina-Montes et al., 2021). Moreover, the prevalence of PDA is low in the LSDM group, and the PDA risk is similar to other common cancers.

But the situation is totally changed in the new-onset diabetes mellitus (NODM) group, which is believed to be a high-risk signal of PDA. NODM is more likely to be a clinical manifestation of PDA and pancreatic precursor lesions because more than 50% PDA related diabetic cases developed diabetes in the 36 months preceding the PDA diagnosis (Setiawan et al., 2019). Moreover, studies also observed a significantly higher PDA risk in the NODM population compared with the LSDM population (Jeon et al., 2018). After removing tumorous tissue, hyperglycemic status and insulin resistance rate were significantly relieved in NODM patients. As for the DM prevalence in cancer, the prevalence of diabetes in the PDA population is significantly higher than noncancer population, whereas DM prevalence in other common cancers is no different from that in normal controls (Aggarwal et al., 2013). Therefore, the relationship between DM and PDA is close and peculiar according to epidemical and clinical studies.

As the first-line antidiabetic drug, insulin is essential for most diabetic patients to control blood glucose. Based on this, the assessment of PDA risk in insulin users is critical, and many studies have been done previously to solve this problem. For long-standing diabetes patients, a relatively low PDA risk population, insulin use is safe and long duration of insulin use is associated with a decreased PDA risk. (Bosetti et al., 2014; Molina-Montes et al., 2021). Unlike other types of cancer, PDA risk in insulin users does not increase with a longer insulin duration. PDA risk in short-term insulin use (less than 3 years) is significantly increased, while people who started insulin more than 5 years showed a relatively lower risk (0.5–0.7 folds) than new starters (Li et al., 2009; Colmers et al., 2012; Singh et al., 2013; But et al., 2017). Laboratory evidence also confirmed that single insulin stimulation could not initiate PDA under low PDA risk circumstances. In primary human pancreatic ductal cells, increased insulin concentration neither enhanced cell survives nor induced cell proliferation (Chan et al., 2014). Knocking out KRAS (the most common gene mutation in 95% PDA patients) in cancer cells even made insulin lose the ability to promote cancer cell viability, and insulin even turned to induced tumor cell dormancy *in vitro* experiments (Rajbhandari et al., 2017). With the introduction of the mutant KRAS gene, insulin regained the ability to induce invasion and proliferation in pancreatic ductal cells (Wang et al., 2019). Based on this, the tumorigenic effect of insulin is primarily driven by mutated cancer genes. Insulin injection or endogenous hyperinsulinemia is safe in long-duration diabetic patients.

However, the situation is changed in high-risk PDA patients. According to the American gastroenterology association (AGA) guideline, the high-risk population of PDA consisted of family history, genetic mutation carriers (including BRCA1, BRCA2, STK11, CDKN2A, etc.), and the NODM population (Aslanian et al., 2020). Although currently there is no direct evidence demonstrating the risk of insulin in initiating PDA in genetic mutation carriers and family history, the activation of insulin signal pathway and circulating hyperinsulinemia elevated the cancer risk in mutated gene carriers (Ding et al., 2012; Argirion et al., 2017). Moreover, hyperinsulinemia predicts PDA in new-onset diabetes mellitus (NODM) patients. As we have discussed before, NODM may be an early symptom of pancreatic precursor lesion or pancreatic cancer. For NODM patients, insulin use or high insulin receptor expression in tumor cells is associated with higher malignancy rates in pancreatic precursor lesions. Thus, insulin accelerates the malignancy of PDA precursor which manifests as a highly increased PDA risk in NODM insulin users (Li et al., 2009; Colmers et al., 2012; Singh et al., 2013; But et al., 2017). Especially in diabetic patients who initiated insulin use less than 0.5 years had the highest PDA risk in short-term cohorts (Colmers et al., 2012; Zanders et al., 2018). As for diabetic PDA patients, insulin is a definite tumor-promoting factor. The mechanism of how insulin influences PDA progression will be discussed later.

## Hyperinsulinemia: A Product Caused by PDA

**FIGURE 1:**
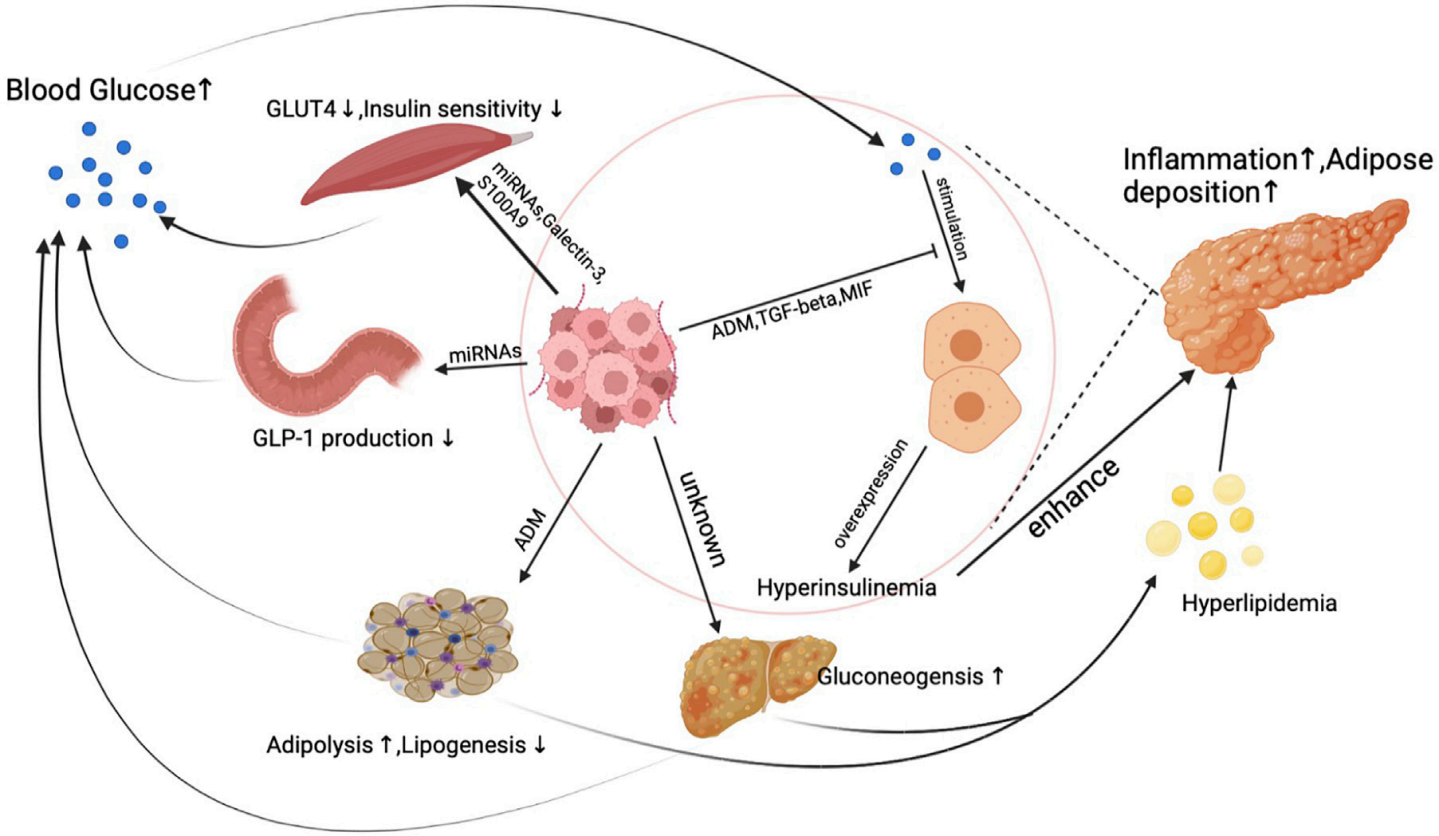
The mechanism of hyperinsulinemia and insulin resistance induced by PDA.

Unlike other cancer, a unique phenomenon is that PDA actively induces hyperglycemia or even diabetes through inducing peripheral insulin resistance as early as the PanIN (a precursor of pancreatic cancer) period. PDA does not directly stimulate insulin overexpression at pancreatic islet beta cells but synthesizes products to elicit insulin resistance in the human body. Reduced insulin sensitivity was observed in pancreatic cancer patients’ muscle, adipose, and liver tissue, which is believed to result from exosomes secreted from pancreatic cancer cells (Basso et al., 1997; Record et al., 2014; Wang et al., 2017). Figure 1. demonstrates the primary mechanism of how PDA induces insulin resistance and blunts glucose sensitivity to cause hyperinsulinemia.

Under physical conditions, insulin stimulates insulin receptors in muscle, adipose, and liver tissue to transport blood glucose into myotube cells, adipocytes, and hepatic cells. Under pancreatic cancer conditions, micro-RNAs (miRNAs) secreted from PDA activate PI3K/Akt/FoxO1 signal pathway to suppress myotube cells’ response to insulin in muscle tissue (Wang et al., 2017). Aside from miRNAs, pancreatic cancer cell generates galectin-3 and S100A9 to suppress glucose up-taking ability in myotube cells (Liao et al., 2019).

Moreover, adipose tissue is also the target of pancreatic cancer. In the pancreatic cancer microenvironment, pancreatic cancer cells and fibroblasts produce adrenomedullin to inhibit adipocyte response to insulin signals. Despite interfering with insulin response in human muscle and adipose tissue, the synthesis of glucagon-like peptide-1 (GLP-1), a well-known glucose-lowering hormone, can also be influenced by pancreatic cancer cells. miRNAs released from exosomes inhibit PCSK1/3 expression by suppressing the production of GLP-1 and glucose-dependent insulinotropic peptide (GIP) in enteroendocrine cells, which finally leads to increased insulin resistance (Valerio et al., 1999; Cases et al., 2015; Zhang et al., 2018). PDA also induces reduced insulin sensitivity in hepatic cells, which represents as a decreased liver gluconeogenesis level (Basso et al., 1997). When a delayed and blunted insulin response happens in PDA patients, elevated blood glucose levels then stimulate insulin synthesis and secretion in pancreatic islet beta cells, leading to endogenous hyperinsulinemia. What is more, because of the high insulin resistance rate, a worsened glucose control was found in diabetic PDA patients who take insulin as a regular regimen to control blood sugar. Increased insulin resistance caused by PDA will subsequently result in a higher dose of insulin use, leading to increased exogenous hyperinsulinemia.

Interestingly, hyperinsulinemia caused by pancreatic cancer might not effectively lower peripheral blood glucose levels. PDA produces multiple factors to inhibit glucose sensitivity in pancreatic islet beta cells which finally leads to a delayed insulin secretion peak (Tan et al., 2014; Parajuli et al., 2020; Wang et al., 2020). As a result, hyperinsulinemia couldn’t effectively control blood glucose levels in pancreatic cancer patients. A dual increase of glucose and insulin is typical in PDA patients and is essential for cancer development and progression.

## Mechanism of PDA Promoting Effect by Insulin

**FIGURE 2:**
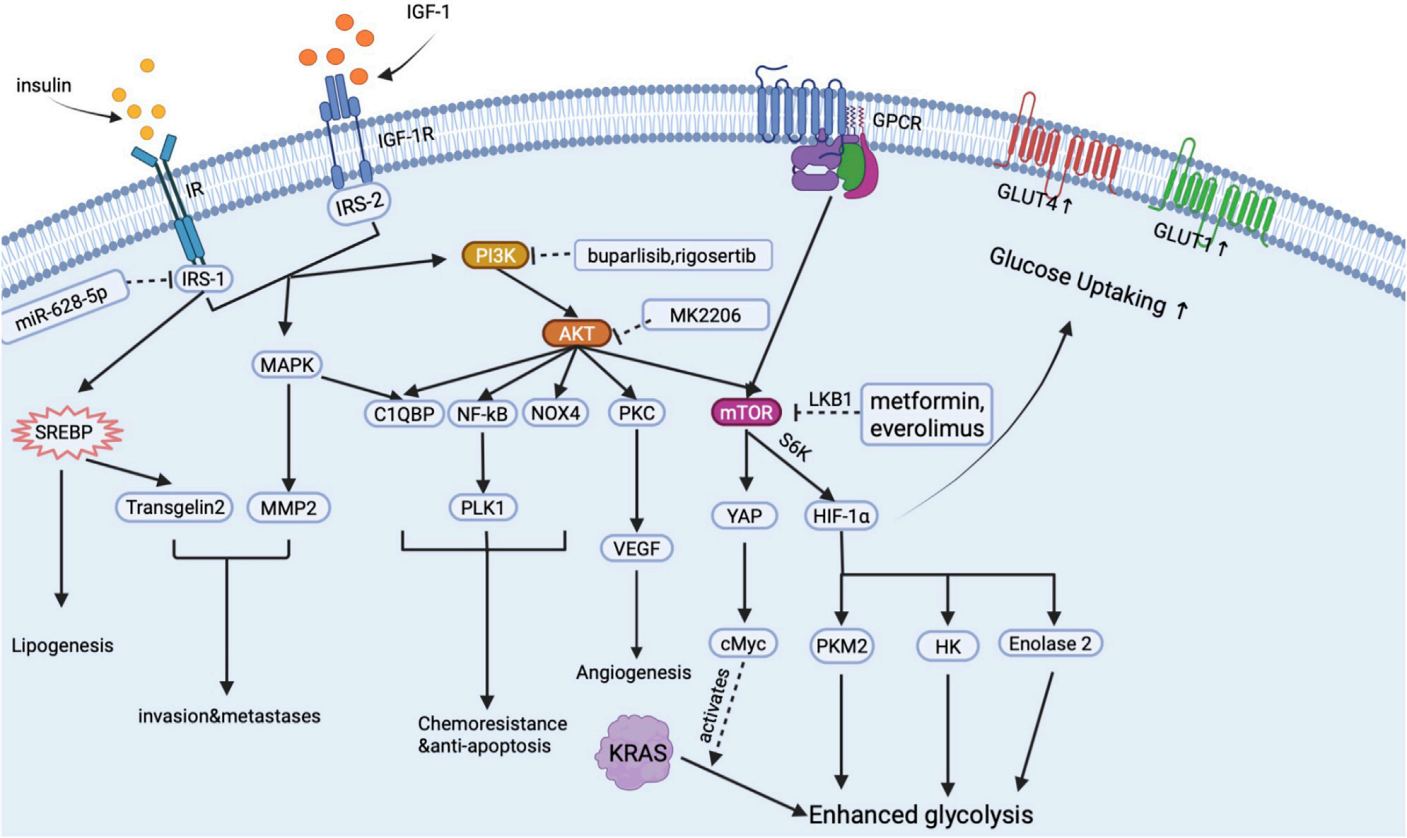
Signal pathways activated by insulin in pancreatic cancer cells.

Insulin has long been regarded as a promoter for cancer metabolism and proliferation in many types of cancer, including breast, prostate, colorectal and pancreatic cancer (Wang et al., 2007). There is a causal relationship between hyperinsulinemia and pancreatic cancer events under an inflammatory environment (Gao et al., 2015). High insulin receptor expression was found in pancreatic cancer tissue, and a high circular insulin level often predicts poor prognosis in PDA cases (Kim et al., 2020; Heckl et al., 2021). Mechanically, insulin mainly activates two signal pathways: insulin receptor (IR)-insulin receptor substrates (IRS)-phosphatidylinositol 3-kinase (PI3K)-protein kinase B (AKT) signal pathway (Bergmann et al., 1996; Pollak, 2008); and extracellular signal-regulated kinase (ERK) 1/2- mitogen-activated protein kinase (MAPK). Based on these two signal pathways, insulin enhances PDA development through mediating metabolic reprogram, crosstalk with IGF-1, strengthening drug resistance, and stimulating tumor microenvironment (inflammation, fibrosis, and neo-angiogenesis) formation. By targeting insulin, PI3K-AKT signal pathway and chemoresistance are inhibited, and the development of PDAC is also inhibited (Metz et al., 2016; Xu et al., 2018). And we summarized the main signal pathway activated by insulin in pancreatic cancer cells in Figure 2.

### Inducing PDA Initiation

Hyperinsulinemia induces chronic inflammation in the microenvironment of pancreatic ductal cells through recruiting fat deposition in pancreatic tissue and stimulating pancreatic fat tissue to secrete inflammatory factors under T2DM circumstances. The mass of pancreatic fat (PF) is directly associated with the production of insulin (Cohen et al., 2014). Under diabetic and pancreatitis circumstances, the proinflammatory effect of hyperinsulinemia is significantly magnified and promoted (Yamazaki et al., 2018; Singh et al., 2019; Zhang et al., 2021). Mechanically, hyperinsulinemia stimulates liver fat accumulation, which secretes free fatty acid into blood vessels and conducts the deposition of the free fatty acid in the pancreas (Pinnick et al., 2008; Gao et al., 2015). Moreover, insulin promotes adipocytes proliferation by stimulating the expression of several factors such as neuropeptide Y (Yang et al., 2008). Under the stimulation of insulin, pancreatic fat consistently secretes inflammatory factors such as IL-6, TNF to alter chronic inflammation in the pancreas, which finally leads to oncogenic mutation and the onset of PDA (Klöting and Blüher, 2014; Murphy et al., 2018; Brocco et al., 2020; Luo et al., 2021). Despite inducing inflammation, the oncogenic mutation can be maintained by insulin in pancreatic precursor lesions (Zhang et al., 2019).

### Mediating Glucose and Lipid Metabolic Reprogramming

By using pO_2_ histography, PDA was reported to be the most hypoxic cancer compared with other types of cancer (Vaupel et al., 2007). A significantly increased aerobic glycolysis level was observed in pancreatic cancer, especially under severe hypoxic situations (Amendola et al., 2019). As we all know, an obvious sign of malignant tumor cells is that they have an increased rate of glycolysis to supply energy, which is the so-called “Warburg Effect.” Reprogrammed metabolism can also be seen in lipid and other metabolism in pancreatic cancer. This reprogrammed metabolism is associated with invasion and metastasis in PDA, while insulin plays a vital role in assisting PDA metabolic reprogram (Qin et al., 2020).

As is shown in Figure 2, when insulin binds to IR anchored on the surface of the PDA cell, insulin activates the PI3K-AKT signal pathway to upregulate the expression of mammalian target of rapamycin (mTOR) receptor through the crosstalk with G protein-coupled receptors (GPCR) (Shen et al., 2019). mTOR is a key regulator in pancreatic cancer glucose metabolism. Downstream effectors of mTOR including hypoxia-induced factor-1 (HIF-1), hexokinase (HK), pyruvate kinase M2 (PKM2), and enolase2 will then directly stimulate aerobic glycolysis and inhibit oxidative phosphorylation in cancer cells, thus leading to the metabolic reprogramming in PDA tissue (Iqbal et al., 2013; Yang et al., 2019; Zheng et al., 2020). Moreover, through the crosstalk with GPCR, insulin activates yes associated protein (YAP) and cMyc to mediate the altered glycolysis induced by KRAS mutation (Hao et al., 2017; Eibl and Rozengurt, 2019). Glucose up-taking ability is also increased with the overexpression of glucose transporters. Insulin stimulates the MAPK signal pathway to increase glucose transporter-1 (GLUT-1) expression and promote the molecular cloning of GLUT-4 (Ding et al., 2000; Sayem et al., 2018). Increased GLUTs then help cancer cells intake enough glucose to support the glucose supply of aerobic glycolysis (Ding et al., 2000). As a result, insulin assists cancer cells to possess a relatively higher glucose up-taking efficiency than normal cells, which may help cancer cells gain advantages in survival competitions. Elevated glycolysis and GLUTs, on the one hand, significantly increase energy supply in cancer cells and, on the other hand, enhance cancer cells’ survival ability in an oxygen-deprived environment, which finally exacerbates the progression of PDA.

Insulin also conducts lipid metabolic reprogram, which manifests as an increased lipid synthesis rate in tumor cells. During this period, insulin promotes lipid synthesis by mediating the expression of sterol regulatory element-binding protein (SREBP)-1 in PDAC cells (Swierczynski et al., 2014; Sun et al., 2017). SREBP-1 then promotes lipid synthesis by increasing fatty acid synthase in cancer cells and mediating transgelin-2 overexpression to increase PDA’s ability to survival and proliferation (Sun et al., 2017; Sunami et al., 2017).

### Stimulating Regional Invasion and Metastases

When treated with Insulin, PDA cells become a more aggressive type that is easier to metastasis and local invasion (Hirakawa et al., 2016). This aggressive PDA is caused by inducing epithelial-mesenchymal transition (EMT) and upregulating matrix metalloprotease-2 (MMP-2) expression to enhance the motility of PDAC cells (Zelenko et al., 2016; Wang et al., 2019). By blocking IR/IGFR signal pathway, the motility and metastases of cancer cells were significantly inhibited (Wang et al., 2019). Moreover, MEK/ERK signal pathway activated by insulin, which can also be triggered by KRAS mutation, plays an essential role in PDA malignancy and invasion (Wang et al., 2019).

### Crosstalk With IGF-1 in Overcoming Apoptosis and Chemotherapies

Insulin and insulin-like growth factor-1 (IGF-1) are two different growth factors synthesized and produced in various organs. Both the receptor of insulin and IGF-1 belong to the receptor tyrosine kinase family. Due to the similar signal pathways activated by Insulin and IGF-1, these two hormones have a similar function in pancreatic cancer. Previously, insulin was often regarded as an alternative factor of IGF-1 in cancer development. However, recently, the crosstalk between Insulin and IGF-1 is raising great attention. Firstly, both Insulin and IGF-1 could promote each other’s expression. In the pancreatic cancer tissue, the tumor-associated fibroblast (TAF) continuously produces IGF-1, which stimulates pancreatic islet beta cells to proliferate and secrete more insulin through a bidirectional microcirculation system in the pancreas (Dheen et al., 1996; Yang et al., 2002; Dybala et al., 2020). Meanwhile, hyperinsulinemia stimulates human liver tissue to synthesize more IGF-1 into blood vessels and enhances the survival and proliferation of TAF. These reciprocating effects indicate a close relationship between Insulin and IGF-1. Secondly, although the main signal pathways activated by insulin and IGF-1 are the same, PI3K-AKT and MAPK-ERK signal pathways, insulin and IGF-1 have been reported to alter different gene expressions in cellular metabolism (Entingh-Pearsall and Kahn, 2004). Studies revealed that insulin has a more substantial effect on the survival of cancer cells, while IGF-1 was better in the cell cycle and proliferation (Cai W. et al., 2017). The crosstalk between Insulin and IGF-1 sustains a balance between cell survival and proliferation in PDA. And the cooperation between Insulin and IGF-1 helps pancreatic cancer resist apoptosis and chemotherapies.

Chemotherapy is an important therapeutic strategy for PDA patients. Many chemical drugs applied in clinical practice kill pancreatic cancer cells by inducing apoptosis (Buck et al., 2010). However, chemotherapy only received a limited benefit: an increase in overall survival of 5 months on average. Our previous study reported that insulin/IGF-1 stimulates Complement component 1, q subcomponent binding protein (C1QBP), to mediate anti-apoptosis and invasion of pancreatic cancer (Shi et al., 2017). Aside from C1QBP, insulin/IGF-1 can also alter the nuclear factor-kB (NF-kB) signal pathway, Polo-like kinase 1 (PLK1), NADPH oxidase 4 (NOX4), and mammalian target of rapamycin (mTOR) to reverse apoptosis, repair DNA and induce multi-drug resistance gene (Lee et al., 2007; Edderkaoui et al., 2011; Choudhury et al., 2015; Soares et al., 2015; Su, 2018). As a result, insulin/IGF-1 causes decreased chemical drugs sensitivity in PDA cells.

Thus, in order to relieve the chemo-resistant effect by IGF-1, IGF-1 receptor (IGF-1R) inhibitors combined with chemical drugs have been applied to treat PDA in clinical trials. However, in clinical trials, the combination of gemcitabine with IGF-1R inhibitor received little overall survival benefits in advanced pancreatic cancer patients (Philip et al., 2014; Fuchs et al., 2015). Laboratory studies revealed that, under an IGF-1 inhibited environment, insulin works as the backup line for cancer cells to gain chemoresistance and resist IGFR related therapies in PDA cells. When treated with an IGF-1 inhibitor, a compensatory insulin receptor (IR) overexpression was observed in pancreatic cancer cells. And the pancreatic islet inside PDA tissue will produce more insulin to nourish cancer cells (Hu et al., 2020). The increased insulin density in the pancreatic cancer microenvironment and IR overexpression in cancer cells finally lead to the failure of IGF-1R inhibitor in pancreatic cancer trials (Buck et al., 2010; Ulanet et al., 2010; Garofalo et al., 2011). The dynamic balance between IR and IGF-1receptor greatly enhances cancer cells’ ability to endure chemotherapies targeting IGF-1R and cancer cell apoptosis.

### TME Inflammation, Fibrosis and Neo-Angiogenesis

The pancreatic tumor microenvironment (TME) plays a critical role in cancer metabolism, metastasis, and drug resistance, which is also the target of insulin. Insulin promotes PDA malignancy and progression *via* influencing fibrosis, angiogenesis, and inflammation in TME.

(1) Enhancing inflammation through elongating the half-life of TAM. Aside from inducing fat deposition to cause inflammation in TME, macrophages living in the TME are also the target of insulin. TAM plays a pivotal role in the progression, chemoresistance, and immune resistance in PDA (Ireland et al., 2016; Griesmann et al., 2017; Wang et al., 2018). Mutant KRAS induces the monocyte to become the tumor-promoting phenotype (M2 type) through secreting many factors to elicit polarization in macrophages (Bishehsari et al., 2018; Dai et al., 2020). However, the half-life of tumor-associated macrophage (TAM) is relatively short. Coincidently, insulin could activate the receptor tyrosine kinases (RTK) signal pathway in TAM and thus inhibit caspase and increase the survival in PDA (Song et al., 2016).
(2) Fibrosis. An extremely dense fibrotic microenvironment caused by fibroblasts is a unique signal of pancreatic cancer, which helps PDA escape from immune therapy and chemotherapy. Insulin has promoted fibrosis in tumor stroma even since the PanIN period (Zhang et al., 2019). With the decrease of hyperinsulinemia, PDA stroma is thinner (Zhang et al., 2019). Insulin directly stimulates tumor-associated fibroblasts (TAF) through activating the IRS-PI3K-AKT-mTOR signaling pathway and inactivating the FoxO1 signal pathway (Yang et al., 2016; Cai C. X. et al., 2017). As a result, insulin removes the growth inhibition of TAF, which is essential for enhancing fibrosis in TME. What is more, insulin stimulates PDA cells to secret Shh (sonic hedgehog), IL-6, tissue growth factor (TGF) to promote fibroblast cell growth in TAM (Mutgan et al., 2018). Interestingly, pancreatic cancer is a type of nutrition-poor cancer. The fibroblasts can also work as an energy supply for the growth of pancreatic cancer. So, the enhanced survival and proliferation caused by insulin are essential for the fibrotic microenvironment formation and the development of cancer cells.
(3) Angiogenesis. Endothelial cells on the micro-vessel are also the target of insulin (Heckl et al., 2021). Insulin stimulates local angiogenesis, which is essential to help cancer cells get more oxygen and nutrients from the blood vessels. Insulin also enhanced angiogenesis by promoting the expression of vascular endothelial growth factor (VEGF) in cancer cells (Stoeltzing et al., 2003; Neid et al., 2004).

## Clinical Advances of Targeting Insulin and Insulin Signal Pathways

In diabetic PDA patients, hyperglycemia and high insulin level are both signals for poor prognosis, but insulin is an essential drug in regulating blood glucose levels (Tseng, 2013; Cho et al., 2019). Singly reducing insulin dosage will cause worse blood glucose control that may kill PDA patients. Two ways to inhibit insulin-related tumorigenic effect with good glucose control are applied in clinical trials and clinical practice: the first is using other safe antidiabetic drugs to lower blood insulin concentration and blood glucose simultaneously; the second is designing new drugs targeting insulin-related signal pathways.

### Alternative Antidiabetic Drugs Selection

In the first method, selecting alternative antidiabetic drugs to decrease insulin dosage requires evidence of the drugs’ safety and potential PDA risk. Luckily, a lot of works about assessing the potential PDA risk of every antidiabetic drug were done previously. The result showed antidiabetic medications such as metformin, SGLT-2 inhibitor, and GLP-1 receptor agonist have anticancer effects while decreasing insulin levels simultaneously.

(1). Glucagon-like peptide-1 (GLP-1) receptor agonist decreases blood glucose by promoting energy storage in adipose tissue and inhibiting glucagon secretion. GLP-1 agonist may help reverse insulin resistance induced by pancreatic cancer cells, thus leading to a reduced blood insulin level (Zhang et al., 2018). As we discussed before, PDA targets GLP-1 production to induce insulin resistance. Targeting GLP-1 is important in relieving hyperinsulinemia caused by PDA cells. Moreover, GLP-1 agonist such as liraglutide activates cAMP that consequently inhibits the AKT signal pathway in pancreatic cancer cells (Zhao et al., 2014a; Zhao et al., 2014b). By suppressing AKT signal pathway, the main signal pathway activated by insulin, GLP-1 may reverse the tumor promoting effect by insulin in pancreatic cancer.
(2). Metformin, an activator of AMPK and LKB1, could decrease hepatic tissue insulin resistance, and prolonged survival was observed in cancerous metformin users (Sadeghi et al., 2012; Amin et al., 2016). Metformin stimulates LKB1 activation to disrupt the crosstalk between IR and GPCR in pancreatic cancer, thus causing decreased cancer proliferation and fibrosis. Studies also revealed that metformin could stimulate the AMPK signal pathway, inhibited by high-glucose status, to induce ferroptosis and apoptosis of pancreatic cancer. However, a recent study indicated that AMPK could phosphorate 3-phosphoglycerate dehydrogenase to enhance the ability to sense cellular energy status and flexibly utilize the available substrate in pancreatic cancer (Hopkins et al., 2018). In clinical, a prolonged survival by metformin was only observed in diabetic PDA patients and no effect was found in the whole PDA population, which indicates the major effect of metformin is decreasing insulin resistance rather than anticancer effect (Chaiteerakij et al., 2016).
(3). Another antidiabetic drug, sodium-dependent glucose transporter-2 (SGLT-2) inhibitor, showed the ability to inhibit the progression of PDA. The overexpression of SGLT-2 was observed in pancreatic cancer cells (Scafoglio et al., 2015). By blocking SGLT-2, a reduced PDA risk was found in SGLT-2 inhibitor users, and a decreased cancer proliferation in preclinical pancreatic cancer models when treated with SGLT-2 inhibitor (Dicembrini et al., 2019; Dąbrowski, 2021).

### Designing New Drugs Targeting Insulin Signal Pathways

Moreover, the insulin signal pathway can be the new druggable target for inhibiting PDA progression. Targeting insulin receptors and downstream factors such as PI3K, mTOR, HIF-1 may help us inhibit tumor progression and restore drug sensitivity in pancreatic cancer. Drugs aimed at mTOR (everolimus, metformin), AKT (MK2206), PI3K (buparlisib, rigosertib) are undergoing phase1-3 clinical trials in advanced pancreatic cancer patients (Wolpin et al., 2009; Ma et al., 2012; Bedard et al., 2015; Chung et al., 2017). Although these drugs got good grades in laboratory and preclinical studies, most failed to inhibit PDA progression and elongate patients’ survival in clinical trials. Currently, drugs treating insulin-related signal pathways receive poor clinical outcomes, which are due to drug intolerance and alternative signal pathway that drive pancreatic cancer cells to survive and proliferation (Chung et al., 2017). More importantly, most of these inhibitors can cause hyperglycemia, a profound off-target effect, leading to drug intolerance and promoting tumor growth in pancreatic cancer patients.

Fortunately, linsitinib (OSI-906), a novel dual IR/IGF-1R inhibitor, showed a good effect in treating pancreatic cancer. Preclinical studies found that linsitinib significantly decreased glycolysis and metastasis in pancreatic cancer. And clinical trials of linsitinib showed a prolonged overall survival of in advanced high IGF-1R expression solid tumors (including lung cancer, melanoma, pancreatic cancer, and gastrointestinal stromal tumors) (Jones et al., 2015; Puzanov et al., 2015; Leighl et al., 2017; von Mehren et al., 2020). Especially in mutant KRAS tumors, linsitinib reached a significantly increased disease stabilization response in clinical trials (Jones et al., 2015). Less than 2% of cases were reported hyperglycemia in linsitinib trials, which means it is safe for glucose management in diabetic pancreatic cancer patients. Novel inhibitors targeting insulin-related signal pathways, including ONC201 (AKT inhibitor), LY294002 (PI3K inhibitor), received satisfying results in preclinical studies (Fujiwara et al., 2008; Zhang et al., 2016). However, single using insulin signal pathway inhibitors can be interfered by hyperinsulinemia. A recent study confirmed that circulating insulin decreased the efficacy of PI3K inhibitors. When treated with insulin lowering methods, including SGLT-2 inhibitor and the ketogenic diet, the tumor cells had a better response to PI3K inhibitors (Hopkins et al., 2018). Combining dual IR/IGF-1R inhibitor with PI3K/AKT/mTOR/HIF-1 inhibitors may help these drugs overcome drug resistance induced by Insulin and IGF-1. Extensive understanding of the dynamic mechanism of how PDA regulates insulin-related signal pathways is needed in the future.

## Conclusion

Over a long period, insulin has been regarded as a compensatory hormone of IGF-1 in cancer development and progression. But recently, more and more studies indicated that insulin might have an independent effect on cancer initiation and metabolic regulation. The relationship between insulin and pancreatic cancer is complex and important. Pancreatic cancer initiates hyperinsulinemia and delays insulin secretion peak, while insulin mediates cancer cell metabolism to maintain PDA survival with proliferation, and insulin also stimulates the formation of a tumor-promoting microenvironment around cancer cells. This reciprocal relationship between cancer and insulin reveals that insulin plays an essential role in pancreatic cancer onset and development. The intricate relationship between pancreatic cancer and insulin allows us to have a better understanding of the PDA initiation, metabolic reprogramming, development, chemoresistance, and metastasis.

Due to the low prevalence of pancreatic cancer in the whole population, insulin is safe for most diabetic patients. In contrast, insulin use should be more careful in high PDA risk people, especially NODM population, and PDA patients. More importantly, the tumor-promoting effect of insulin in pancreatic cancer cells and the TME is essential, which makes insulin and its related signal pathway a potential novel target for cancer therapies. Lowering peripheral insulin level *via* using other antidiabetic drugs is feasible, but therapies targeting insulin signal pathways still needs a long way to go at present. Cancer could use insulin to compensatorily activates multiple signal pathways to escape from IGF-1R inhibitors. This mechanism indicates that combining various drugs aimed at different targets in the insulin signal pathway with dual IR/IGF-1R inhibitor may be an excellent way to overcome drug resistance caused by alternative insulin related pathways activation.

## Abbreviations

AGA, American gastroenterology association; AKT, protein kinase B; PDA, pancreatic cancer; DM, diabetes mellitus; LSDM, long-standing diabetes mellitus; NODM, new-onset diabetes mellitus; IR, insulin receptor; IRS, insulin receptor substrates; PI3K, phosphatidylinositol 3-kinase; ERK, extracellular signal-regulated kinase; MAPK, mitogen-activated protein kinase; PF, pancreatic fat; GPCR, G protein-coupled receptors; mTOR, mammalian target of rapamycin; HIF-1, hypoxia-induced factor-1, HK, hexokinase, PKM2, pyruvate kinase M2; YAP, yes associated protein; GLUT-1, glucose transporter-1; GLUT-4, glucose transporter-4; SREBP)-1, sterol regulatory element-binding protein; MMP-2, matrix metalloprotease-2; IGF-1, insulin-like growth factor-1; IGF-1R, IGF-1 receptor; C1QBP, Complement component 1, q subcomponent binding protein; NF-kB, nuclear factor kB, PLK1, Polo-like kinase 1; NOX4, NADPH oxidase 4; TME, tumor microenvironment; TAM, tumor-associated macrophage; RTK, receptor tyrosine kinases; TAF, tumor-associated fibroblasts; Shh, sonic hedgehog; TGF, tissue growth factor; VEGF, vascular endothelial growth factor; GLP-1, glucagon-like peptide-1; ADM, adrenomedullin; MIF, macrophage migration inhibiting factor; GLUT2, glucose transporter-2; SGLT-2, sodium-dependent glucose transporter-2.
